# The Paradigm of G Protein Receptor Transactivation: A Mechanistic Definition and Novel Example

**DOI:** 10.1100/tsw.2011.75

**Published:** 2011-03-22

**Authors:** Peter J. Little, Micah L. Burch, Sefaa Al-aryahi, Wenhua Zheng

**Affiliations:** ^1^ Discipline of Pharmacy, School of Medical Sciences, RMIT University, Melbourne, Victoria, Australia; ^2^ Diabetes Complications Group, Health Innovations Research Institute, RMIT University, Melbourne, Victoria, Australia; ^3^ Diabetes and Cell Biology Laboratory, BakerIDI Heart and Diabetes Institute, Melbourne, Victoria, Australia; ^4^ Monash University, Departments of Medicine and Immunology, Central and Eastern Clinical School, Alfred Health, Melbourne, Victoria, Australia; ^5^ Neuropharmacology, School of Pharmaceutical Sciences, Sun Yat-Sen University, Guangzhou, Guangdong, China

**Keywords:** G protein–coupled receptors, protein kinase receptors, transactivation, and definition

## Abstract

Seven transmembrane G protein—coupled receptors are among the most common in biology and they transduce cellular signals from a plethora of hormones. As well as their own well-characterized signaling pathways, they can also transactivate tyrosine kinase growth factor receptors to greatly expand their own cellular repertoire of responses. Recent data in vascular smooth muscle cells have expanded the breadth of transactivation to include serine/threonine kinase receptors, specifically the receptor for transforming growth factor beta (TGF-β). Stimulation with endothelin and thrombin leads to the rapid formation of C-terminal phosphorylated Smad2, which is the immediate product of activation of the TGF-β receptor. Additionally, it appears that no definition of transactivation based on mechanism is available, so we have provided a definition involving temporal aspects and the absence of de novo protein synthesis. The phenomenon of transactivation is an important biochemical mechanism and potential drug target in multiple diseases.

G protein–coupled receptors (GPCRs) are one of the largest families of receptors in biology and are a major category of targets for therapeutic agents[1,2]. GPCR signaling, both at the receptor level and downstream pathways of GPCRs, has been the subject of intense research over several decades and multiple pathways have been described[3,4,5,6]. One of the most interesting pathways involves the transactivation of growth factor receptors, specifically protein tyrosine kinase (PTK) receptors, the activation of which immensely expands the range of cellular functions attributable to GPCRs[6,7,8].

Downstream pathways include GPCR activation of phospholipase C, and the generation of inositol 1,4,5-trisphosphate ($IP_3$) and diacylglycerol[9] and transactivation of receptor tyrosine kinases[10]. Using angiotensin II (AII) receptor activation in vascular smooth muscle cells and responses in blood vessels as an example, the functional consequences of the multiple downstream signal pathway are the production of $IP_3$ leading to the immediate release of calcium from the sarcoplasmic reticulum and acute regulation of vascular tone, a response occurring in seconds[11]. The generation of diacylglycerol leads to the activation of protein kinase C and a cascade leading to the phosphorylation of proteins associated with vascular contraction and the maintenance of a medium-term contraction in a process extending over hours[12,13]. In 1996, Ulrich and colleagues discovered that a GPCR agonist such as AII could lead to the "transactivation" of a protein tyrosine–coupled receptor, specifically the epidermal growth factor (EGF) receptor, leading to phosphorylation of EGFR and the generation of products such as phospho ERK, which are downstream of the EGFR[7,8]. This is very important because GPCR agonists are not generally capable of generating a cell growth signal, but the ability to transactivate a fully competent growth factor receptor, such as the EGFR, gives the GPCR the ability to generate a full-blown cell growth response. Since this original demonstration, there have been many examples in which GPCR agonists, including AII, thrombin, and endothelin, can transactivate multiple PTK receptors, including PDGF, FGF, and IGF-1[14,15,16,17,18,19]. The transactivation of tyrosine kinase receptors such as PDGF and EGF leads to the long-term regulation of vascular tone through effects on vascular smooth muscle cell proliferation and the production of extracellular matrix, with these effects occurring over days or longer, representing remodeling that alters the structure of a blood vessel in response to a pathological stimuli[6,7,20,21,22]. Thus, transactivation leading to vascular remodeling is a very important biological process.

Thus far, the paradigm of GPCR signaling involving receptor transactivation has been limited to PTK receptors. There have been many studies since the original observation and multiple distinct mechanisms have been discovered for the transition of the signal from the GPCR to the PTK receptor[10,23]. These processes have involved intracellular signaling pathways such as calcium ions and reactive oxygen species, and an intriguing extracellular pathway in which GPCR occupation leads to the generation and autocrine/paracrine action of heparin-binding EGF (HB-EGF), which activates EGFRs[24,25]. All of these distinct processes have been included under the term receptor "transactivation".

We have recently discovered that two GPCR agonists can independently activate their respective GPCRs, leading to the generation of C-terminal phosphorylated Smad transcription factor (phosphoSmad2C or pSmad2)[26,27], which is the immediate downstream product of activation of the serine/threonine kinase activity of the transforming growth factor beta (TGF-β) receptor (TβRI), also known as activin-like kinase V (ALK V)[28,29]. The generation of phosphoSmad2C is blocked by both inhibitors of the primary GPCR: bosentan for endothelin receptors, and JNJ5177094 or SCH 79797 for protease activated receptors (PAR)–1 for thrombin[28,29]. Critically, both responses are also blocked by the widely used and selective ALK V inhibitor SB431542[28,29,30]. This pathway fits the use of the term "transactivation" as used in the literature relating the GPCR activation of receptors. There have been several examples of this GPCR to S/TK receptor transactivation where the authors have not used the term transactivation in their descriptions of the interaction[31,32,33,34].

There have been several explanations or definitions of the term transactivation in the context of receptor to receptor transactivation, but none that extended to an inclusion of the mechanism of the interaction. Berry and colleagues provided the first definition as simply "the process whereby ligand stimulation of one receptor leads to activation of another, distinct receptor"[35]. Wetzker and Bohmer[36] offered an explanation of transactivation as being when one receptor transactivates a heterologous receptor. It should be noted that receptor transactivation as considered here is distinct from the transactivation of genes by transcription factors[36]. We thus propose the mechanistic definition that receptor transactivation is "the agonist occupancy of its cognate GPCR complex which leads in a relatively short time and in the absence of '*de novo*' protein synthesis to the activation of and cytosolic generation of the immediate downstream product(s) of a second cell surface protein kinase receptor" (Fig. 1). Obviously, it is necessary to add a conceptual component that takes into account the temporal characteristics

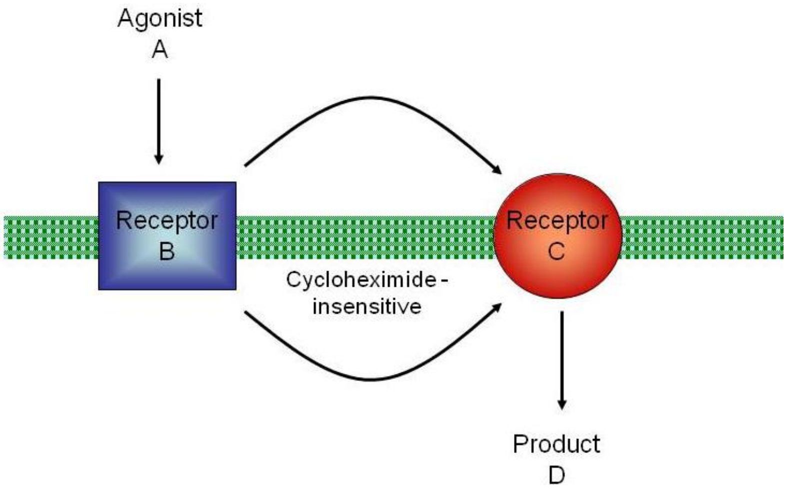

**FIGURE 1.** Model of GPCR receptor to protein kinase receptor transactivation. An agonist A of a receptor B, in this case a GPCR, leads via a mechanism, which is insensitive to the protein synthesis inhibitor cycloheximide, to the activation of a second receptor C, in this case a protein kinase receptor, and the formation of the product D, a phosphorylated signaling intermediate often being a transcription factor. The process is required to occur in a relatively short time frame to constitute “transactivation”. A specific new example is given in Fig. 2.

of the response because long-term receptor occupancy can lead to a multitude of secondary and tertiary interactions, and certainly the gene to mRNA to protein sequence should be excluded from the definition of transactivation.

In view of the literature on GPCR transactivation of PTK receptors, the definition should be taken to include all mechanisms that lead to the generation of the index product, and it should include mechanisms that are intra/extracellular and mechanisms that may involve, but also may not involve, a direct interaction between the two receptors of interest. The proposed definition includes the concept of the pathway being not dependent on *de novo* protein synthesis. This arises from consideration of the involvement of transcription and translation in the definition of receptor “transactivation”. Should the occurrence of transcription or translation, meaning in practical terms that the response is sensitive to actinomycin D or cycloheximide, respectively, be interpreted as meeting or not meeting the definition of transactivation? We would suggest that sensitivity to cycloheximide should exclude the pathway from the definition of receptor to receptor “transactivation”. In most cases, it is to be expected that this will also be picked up by the temporal caveat in that a process requiring transcription and translation of a new protein is likely to take many hours and therefore extend beyond the time indicated above to which the use of the term “transactivation" should be applied.

Receptor to heterologous receptor transactivation has implications in the identification of drug targets for various diseases. TβRI stimulates the synthesis of collagen by vascular smooth muscle and other cells (fibroblasts), leading to excess collagen deposition and ultimately organ fibrosis as the eventual pathology[32,37,38]. Accordingly, TβRI appears to be a valid target to prevent the fibrotic actions of TGF-β. However, receptor transactivation raises the possibility that the apparent primary response is hiding or masking a response that is due to receptor transactivation. In our own studies, we have used proteoglycan and glycosaminoglycan synthesis as a functional readout because of its association with increased lipid binding in the context of the initiation of atherosclerosis[39,40,41,42]. The actions of thrombin and endothelin to generate phosphoSmad2C through transactivation of TβRI are fully functional when assessed as the ability to stimulate the synthesis of the proteoglycan biglycan and cause glycosaminoglycan elongation in human vascular smooth muscle cells[28,29]. Hence, it is possible that a GPCR stands behind a growth factor receptor and in the current case, a GPCR agonist such as thrombin or endothelin may be playing a previously unrecognized role in the stimulation of proteoglycan synthesis via the transactivation of TβRI. These pathways warrant full investigation for their occurrence *in vivo* and their potential role in multiple pathologies.

In conclusion, we have recently found that the paradigm of GPCR transactivation can be extended to include the transactivation of the serine/threonine kinase receptors, specifically TβRI (Fig. 2). In the apparent absence of a definition of receptor transactivation involving mechanism, we have provided a definition that includes the response being relatively rapid, and not involving *de novo* protein synthesis as an indication that the response is direct and does not involve the secondary or tertiary generation of new proteins to affect the transactivation. The implications of receptor transactivation are as previously recognized by others[43], that the apparent primary response such as the generation of a phosphorylated transcription factor may not arise solely from the actions of the protein kinase receptor by its cognate ligand, but may involve the earlier activation of a GPCR and transactivation. A further implication is that the therapeutic target may be the GPCR.

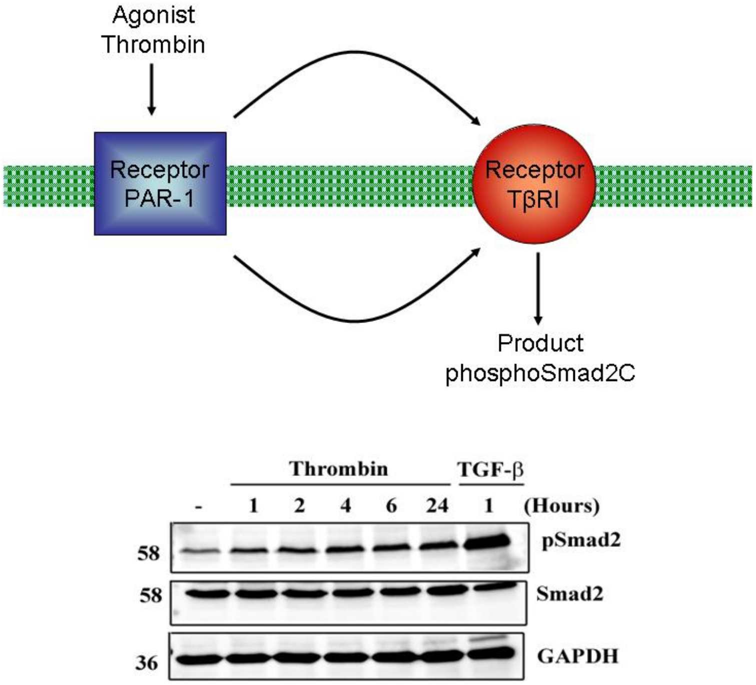

**FIGURE 2.** A novel example of G protein to protein Ser/Thr kinase receptor "transactivation". Although the paradigm has been previously restricted to the GPCR transactivation of PTK receptors, it has recently been reported that GPCRs can transactivate the serine/threonine kinase activity of the TβRI receptor, leading to the rapid formation of phosphoSmad2. The data show that the level of pSmad2C is clearly increased by 1 h, and it is explained in the text that this response is blocked by both inhibitors of the thrombin receptor (PAR-1) and TβRI. (The research shown was originally published in Burch et al.[28]. © The American Society for Biochemistry and Molecular Biology.)
